# Characterization of differential antibody production against hepatitis C virus in different HCV infection status

**DOI:** 10.1186/s12985-016-0572-9

**Published:** 2016-06-30

**Authors:** Mona Rafik, Salwa Bakr, Dina Soliman, Nesrine Mohammed, Dina Ragab, Walid Abd ElHady, Nancy Samir

**Affiliations:** Clinical and Chemical Pathology Department, Faculty of Medicine, Ain Shams University, Children village, POB 9505, Nasr city, Cairo Egypt

**Keywords:** Antibody response, HCV Ag, RIBA

## Abstract

**Background:**

The Centers for Disease Control and Prevention (CDC) issued an update on hepatitis C virus (HCV) testing approach, in which it omitted the use of recombinant immunoblot assay (RIBA) in the diagnostic algorithm and recommended that future studies are needed to evaluate the performance of HCV testing without RIBA. As Egypt has the highest prevalence of HCV worldwide, we aimed to evaluate the value of RIBA in HCV testing in a high prevalence population. Our objective was to clarify whether enzyme linked immunosorbent assay (ELISA) anti-HCV signal-to-cutoff (S/CO) ratios were able to discriminate true positive from false positive anti-HCV antibody status and to evaluate the role of RIBA in solving this problem which may lead to a redefined strategy for diagnosis of HCV infection. Our second objective was to elucidate the effects of different HCV peptides of both structural and non-structural proteins on the humoral immune response to HCV infection.

**Methods:**

The current study drew results from 167 individuals divided into three groups: Group I: included 77 HCV antibody positive (ELISA) high risk health care workers (HCW), Group II: included 56 presumably uninfected individuals who showed normal liver enzymes, negative HCV RNA and were asymptomatic. Their ELISA HCV antibody S/C ratio ranged from 0.9 to <5. Group III: included 34 patients enrolled from outpatient clinics of Ain Shams Hospital with persistent viral replication, elevated liver enzymes, and chronic HCV related liver disease. All study participants were assessed for the presence of anti-HCV antibodies by 3^rd^ generation ELISA which was confirmed by RIBA.

**Results:**

Interpreting the results of both ELISA and RIBA together, false positive results were highly significantly increased in HCW when compared with the other two groups. Indeterminate and false negative results were only found in the presumably uninfected group. For differentiated antibody responses by RIBA, chronic HCV cases had the highest frequency of positive antibody response to core peptides while the presumably uninfected group had the lowest. Antibody response to E2 was found less frequently in chronic cases than Core 1, Core 2 and NS3. The specific antibody response to the different HCV peptides showed the same distribution of frequencies in both chronic HCV cases and the presumably uninfected individuals with the chronic cases having the highest frequencies. This distribution was different from the HCW. The most evident difference was the reaction towards NS3 which was the highest antibody producing peptide in chronic HCV and presumably uninfected individuals whereas in HCW Core1 was the highest.

**Conclusion:**

The HCV antibody immunoblot assay (RIBA) is still necessary for the detection of false positive cases which can occur quite frequently in countries of high prevalence as Egypt. Indeterminate RIBA results indicate a waning antibody response in elderly individuals who recovered from previous or distant HCV infection.

## Background

Hepatitis C virus (HCV) infects >2 % of the world population, with an estimated >500,000 new infections annually in the highest endemic country, Egypt [1]. Although some HCV-infected individuals can resolve infection without drug treatment, ~70 % develop chronic hepatitis and, over a period of 20–30 y, 20–30 % will develop liver cirrhosis and 1–5 % will develop hepatocellular carcinoma [2]. HCV is classified in the *Hepacivirus* genus within the *Flaviviridae* family. The structural HCV proteins include the core protein and transmembrane glycoprotein, E1 and E2 [3]. HCV has six nonstructural proteins; NS2, NS3, NS4A, NS4B, NS5A and NS5B [4].

The humoral response to HCV infection is broadly targeted, with antibodies to both structural and non-structural proteins found in most cases [5]. Although the commercial methodology to detect HCV-specific RNA and antibody responses in patient sera has greatly advanced in recent years, there is no detailed information of the immunogenicity of different HCV proteins in patients suffering from chronic HCV infection [6]. On the other hand, healthy carriers of HCV infection exhibit a specific antibody response against HCV antigens, which could play a role in disease control. Detection of these antibodies may permit a thorough characterization of this response and further identify particular antibodies with potential clinical value [7].

HCV antibody screening tests with enzyme-linked immunosorbent assays (ELISA), were proven to be both highly reliable and cost effective, which led to their almost universal utilization as a first-level screening procedure. However, both *false positive* [HCV-positive according to ELISA, but negative with a second-level recombinant immunoblot assay (RIBA)] and *indeterminate* results (HCV-positive with ELISA, indeterminate results with RIBA) may occur [8]. RIBA is the preferred supplementary serological testing method due to its robust specificity [9].

In this study, our primary aim was to determine the qualitative differences in host antibody responses to different HCV proteins in Egyptian chronic HCV infection and health care workers and their correlation to clinical outcome. Our secondary aim was to assess the need for RIBA testing in a high prevalence setting as found in Egypt to discriminate true positive from false positive anti-HCV antibody status.

## Methods

### Subjects and sample collection

Prior to initiation, this study received approval by the Ethical Committee of the Faculty of Medicine, Ain Shams University. The study included a total of 167 individuals in three groups. Group I: included 77 ELISA HCV antibody positive high risk HCWs (22 males and 55 females) with a mean age of 41.1 ± 10.9 years who worked at Ain Shams University laboratory and blood bank and were thus considered a high risk health care population. Group II: included 56 presumably uninfected individuals (34 males and 22 females) with a mean age of 56.2 ± 12.5 years enrolled from Ain Shams University Hospital who were well characterized asymptomatic patients with normal liver enzymes and negative for HCV RNA. Their ELISA HCV antibody S/C ratio ranged from 0.9 to <5. Group III: This group included 34 patients (17 males and 17 females) with a mean age of 42.5 ± 8.1 years enrolled from outpatient clinics of Ain Shams University Hospital with persistent HCV viral replication, elevated liver enzymes and chronic HCV related liver disease.

Venous blood samples (5 mL) were obtained from all participants. Samples were allowed to clot and sera were then separated by centrifugation (3500 rpm, 20 min, 25 °C) and then stored in aliquots at −20 °C until used for analysis of the various parameters outlined below.

### Measurement of liver enzymes

Serum levels of Alanine aminotransferase (ALT) and aspartate aminotransferase (AST) were determined on a Synchron CX-9 chemistry analyzer (Beckman Instruments Inc., CA, USA). ALT and AST levels below 40 were considered normal.

### Detection of HCV antibodies by ELISA

Presence of Anti-HCV antibodies was assessed using by 3^rd^ generation ELISA (Diasorin S.P.A., Italy). The samples were considered positive for anti-HCV antibodies when the index values (S/CO) were >1.1, non-reactive when values were ≤0.9 and indeterminate when values ranged between >0.9 and 1.1.

### Detection of HCV RNA

Real time polymerase chain reaction was used for detection and quantitation of HCV RNA. HCV RNA was extracted from serum using QIAamp Viral RNA Mini Kit (Qiagen, Duesseldorf, Germany), then, the extract was added to Brilliant QRT-PCR 1-step Master Mix (Stratagene, La Jolla, USA) and real-time RT-PCR was done on Stratagene Mx3000P device.

### Recombinant immunoblot assay

The amount and profile of the HCV antibody was confirmed by the semiquantitative recombinant immunoblot assay (RIBA), using INNO-LIA™HCV Score supplied by INNOGENETICS, Belgium. The INNO-LIA® HCV Score assay utilizes well-defined antigens derived from HCV immunodominant proteins from the core region, the E2 hypervariable region (HVR), the NS3 helicase region and the NS4A, NS4B and NS5A regions. Band reactivity is graded by visual calibration against IgG control bands present on each strip. The intensity of the colored bands is proportional to the amount of bound antibody and is graded as - (none), ± and 1+ to 4.

A sample was considered positive when at least two HCV bands had reactivity of ± or higher, indeterminate when either a single NS3 band had a reactivity of ± or higher, or any other single band had reactivity of +1 or higher, and a result was considered negative either when no band reactivity was present or when only one HCV antigen line had a reactivity of ±, except when the reactivity was observed for NS3.

### HCV Antibody results’ interpretation

Interpretation of RIBA results with ELISA results was done as follows; sera were considered 1- true positive: anti-HCV antibodies indeterminate or positive by ELISA and positive by RIBA, 2- false positive: anti-HCV antibodies positive by ELISA with either indeterminate or negative RIBA or anti-HCV antibodies indeterminate by ELISA with negative RIBA, 3- indeterminate: anti-HCV antibodies are indeterminate by both ELISA and RIBA and 4- false negative: anti-HCV antibodies negative by ELISA and positive or indeterminate by RIBA.

### Statistical analyses

Statistical analysis was carried out with SPSS statistical software version 22.0 (SPSS Inc., Chicago, IL, USA) for Windows and a *p-*value < 0.05 was considered as significantly different and a *p-*value < 0.01 was considered as highly significant.

## Results

### Descriptive characteristics of study participants

All 167 study participants were assessed for the presence of anti-HCV antibodies by 3^rd^ generation ELISA and confirmed by RIBA. Descriptive statistics of the studied groups is shown in Table 1.

**Table 1:** Descriptive statistics of the studied group

	HCW	Presumably uninfected	Chronic HCV
Age, mean ± SD	41.1 ± 10.9	56.2 ± 12.5	42.5 ± 8.1
Sex (M/F)	22/55	34/22	17/17
ALT (IU/L), Mean ± SD	27 ± 22.5	25.4 ± 11.4	42.1 ± 23.1

### Comparison of ELISA and RIBA results

Out of the total 167 samples, 123 were anti-HCV antibodies positive, 5 were negative and 39 were indeterminate by ELISA. When RIBA testing was performed out of the 123 anti-HCV antibodies positive by ELISA, 96 were positive, 6 were indeterminate and 21were negative.

True positive results were highly significantly increased in chronic hepatitis 91.2 % (31/34), followed by HCW 71.4 % (55/77) then the presumably uninfected group 48.2 % (27/56). False positive cases were highly significantly increased in HCW 28.6 % (22/77) when compared with presumably uninfected 8.9 % (5/56) and chronic 8.8 % (3/34). Indeterminate and false negative results were only present in the presumably uninfected group (Table 2).

**Table 2:** Comparative statistics of HCV antibody status by ELISA and RIBA

	Groups tested, *n*								
	HCW	Presumably uninfected	*p*	Presumably uninfected	Chronic	*p*	HCW	Chronic	*p*
ELISA									
negative	0	5		5	0		0	0	
indeterminate	0	39		39	0		0	0	
positive	77	12		12	34		77	34	
RIBA									
negative	19	4		4	1		19	1	
indeterminate	3	23	<0.001	23	2	<0.001	3	2	0.044
positive	55	29		29	31		55	31	
ELISA + RIBA									
true positive	55	27		27	31		55	31	0.022
false positive	22	5	<0.001	5	3	<0.001	22	3	
indeterminate	0	19		19	0		0	0	
false negative	0	5		5	0		0	0	

Indeterminate RIBA results were observed in 16.8 % (28/167) of the sample submitted to RIBA. Of these 3 samples were detected in HCW, 2 samples were detected in chronic, while 23 sample were detected in the presumably uninfected group. In HCW the reactivity was due to core1, core2 and NS3. In chronic HCV cases, the reactivity was due to NS3. While in the presumably uninfected group the reactivity was due to core1 in 2 samples and core2 in another 2 sample while 19 samples were due to NS3 (Table 3).

**Table 3:** Distribution of reactivity to HCV antigens in the studied group with indeterminate RIBA results

	Core1	Core2	NS3
HCW	1	1	1
Presumably uninfected	2	2	19
Chronic	0	0	2

### Differentiated antibody responses in RIBA testing

Chronic HCV cases had the highest frequency of positive antibody response to both core 1 and core 2 peptides (91.2 %,76.5 %) than both HCW (70.1 %, 68.9 %) and the presumably uninfected group (48.2 %, 46.4 %). Moreover, Core 1and 2 in HCW proved to be highly significantly increased when compared with presumably uninfected group.

Comparison of E2 antibody response between the studied groups revealed a non-significant difference between chronic cases (35.3 %) and HCW (28.6 %). However, there was a highly significant increase in antibody responses to E2 in chronic HCV cases when compared with the presumably uninfected group (8.9 %) and a significant increase in HCW when compared to the presumably uninfected group.

Antibody responses to NS3 were found to be highly significantly increased in chronic HCV cases (97.1 %) when compared with the presumably uninfected group (78.6 %) and with the HCW (65 %). NS3 antibody responses in HCW were found to be highly significantly decreased when compared with the presumably uninfected group. Antibody responses to NS4 were highly significantly increased in chronic HCV cases (67.6 %) when compared with HCW (46.8 %) and with presumably uninfected individuals (16.1 %). Similarly the HCW showed a highly significant increase when compared with presumably uninfected individuals. Comparison between the studied groups as regards the reactivity to NS5 peptides revealed a highly significant increase in chronic HCV cases (44.1 %) when compared to both the presumably uninfected group and HCW. A significant increase in reactivity to NS5 peptides was found in HCW when compared with the presumably uninfected group. To summarize, the antibody response to core (1& 2) and NS4 & NS5 show the same pattern of significantly increased values in chronic HCV cases than both HCW and presumably uninfected group with the HCW being significantly higher than the presumably uninfected group. As for the E2 antibody response there was no significant difference between the chronic HCV cases and the HCW while the presumably uninfected group had significantly lower values than both. The NS3 response showed a significantly lower response in HCW than both chronic HCV cases and the presumably uninfected group while the chronic HCV cases had a significantly higher value than the presumably uninfected group (Table 4).

**Table 4:** Comparative statistics of antibody reactivity to HCV antigens in the studied groups

HCV peptide	Groups tested, *n*								
	HCW	Presumably uninfected	*p*	Presumably uninfected	Chronic	*p*	HCW	Chronic	*p*
Core 1									
negative	23	29	0.003	29	3	<0.001	23	3	<0.001
positive	54	27		27	31		54	31	
Core 2									
negative	24	30	<0.001	30	8	0.001	24	8	<0.001
positive	53	26		26	26		53	26	
E 2									
negative	55	51	0.017	51	22	0.002	55	22	0.29
positive	22	5		5	12		22	12	
NS3									
negative	27	12	<0.001	12	1	0.002	27	1	<0.001
positive	50	44		44	33		50	33	
NS4									
negative	41	47	0.001	47	11	<0.001	41	11	<0.001
positive	36	9		9	23		36	23	
NS5									
negative	56	51	0.018	51	19	<0.001	56	19	0.011
positive	21	5		5	15		21	15	

### Comparison of frequencies of specific antibody response

When we studied the frequencies of the specific antibody response to the different HCV peptides we found that chronic HCV cases and the presumably uninfected individuals had the same distribution of frequencies (NS3, C1, C2, NS4, NS5, and E2) with the chronic cases having the highest frequencies. This distribution of specific antibody responses was different in the HCW (C1, C2, NS3, NS4, E2 and NS5). The most evident difference was the reaction towards NS 3 peptides which was the highest antibody producing peptide in chronic HCV and presumably uninfected individuals whereas in HCW C1 was the highest (Table 5).

**Table 5:** Frequency of specific antibody responses in studied groups

HCW		Presumably uninfected		Chronic	
NS5	27.3 %	E2	8.9 %	E2	35.3 %
E2	28.6 %	NS5	9.0 %	NS5	44.1 %
NS4	46.8 %	NS4	16.3 %	NS4	67.0 %
NS3	65.0 %	C2	46.4 %	C2	76.5 %
C2	68.9 %	C1	48.2 %	C1	91.2 %
C1	70.1 %	NS3	78.6 %	NS3	97.3 %

### Breadth and strength of the antibody response

The breadth and the intensity of the antibody response detected by the number of positive bands observed in RIBA, was classified into 1 band (indeterminate), low intensity (2 bands) and strong intensity(3-6 bands). This revealed that in chronic cases 90.9 % (30/33) presented with reactivity to three antigens or more, while in HCW the reactivity to three antigens or more was 86.2 % (50/58) and in the presumably uninfected group the reactivity to three antigens or more was 13.5 % (21/52) (Table 6). There was no significant difference in the breadth and strength of the antibody response between the chronic HCV cases and the HCW, however both chronic HCV cases and the HCW showed a significantly wider and stronger antibody response than the presumably uninfected group.

**Table 6:** Comparative statistics of the breadth and strength of antibody response observed in RIBA testing in studied groups

	Groups tested, *n*								
	HCW	Presumably uninfected	*p*	Presumably uninfected	Chronic	*p*	HCW	Chronic	*p*
1 band	3	23	<0.001	23	2	<0.001	3	2	0.069
2 bands	5	8		8	1		5	1	
3-6 bands	50	21		21	30		50	30	

### Multiple regression analysis

Multiple regression analysis was carried out to detect which HCV peptides could differentiate chronic from HCW and presumably uninfected individuals. The presence of antibodies against both Core 2 and NS3 in chronic HCV cases can differentiate chronic cases from HCW with Odd^,^ s ratio 12.38 (Table 7). While the presence of antibodies against both Core 1 and NS4 in chronic HCV cases can differentiate them from presumably uninfected individuals with Odd’s ratio 10.33 (Table 8).

**Table 7:** Multiple regression analysis for discrimination to HCV antigens reactivity among HCW and chronic

		HCW	Chronic	*p*	Odd’s ratio
Core 2 + NS3	negative	21	1	0.003	12.38
	positive	56	33

**Table 8:** Multiple regression analysis for discrimination to HCV antigens reactivity among presumably uninfected and chronic HCV subjects

		Presumably uninfected	Chronic	*p*	Odd’s ratio
Core 1 + NS4	Negative	28	3	<0.001	10.33
	Positive	28	31		

### Association of RIBA results with clinical data

#### Age

Comparison of the median age of the RIBA indeterminate (55.5 years) and RIBA positive (44 years) subjects, proved that there was a highly significant difference with RIBA indeterminate showing a higher age (Table 9).

**Table 9:** Comparative statistics of RIBA positive and RIBA indeterminate as regard age

RIBA	Age Median (range)	*p*	Odd’s ratio
positive	44 (35-53)	<0.001	10.33
indeterminate	55.5 (49-65.5)		

### Viral load

Comparative statistics of viral load with number of bands in RIBA testing in chronic HCV patients proved to be non-significant (Table 10). RT-PCR was carried out for a proportion of the true positive and indeterminate HCV antibody results (Table 11). In both the HCW and the presumably uninfected group all PCR results were found negative for both true positives and indeterminate HCV antibody results.

**Table 10:** Comparative statistics of viral load (IU/ml) with number of bands in chronic HCV patients

	Number of patients	Viral load, median (range)	*P*
0 band	1	4850000	0.137
1 band	2	295250 (12500-578000)	
2 bands	1	354000	
3 bands	5	474 (40-184000)	
4 bands	11	134000 (335-4170000)	
5 bands	7	139000 (166-4560000)	
6 bands	7	25000 (2025-249000)

**Table 11:** HCV Real-time RT-PCR results in true positives and indeterminate HCV antibody results

	HCW	Presumably uninfected	Chronic
True positive = 113	55	27	31
RT-PCR (Number of cases)	25	26	34
Positive	0	0	34
Negative	25	26	0
Indeterminate =19	0	19	0
RT-PCR (Number of cases)	0	11	0
Positive	0	0	0
Negative	0	11	0

## Discussion

Hepatitis C virus is a global health problem and the World Health Organization estimates that at least 170 million people are infected with HCV worldwide, with most of these concentrated in developing countries. The high incidence of HCV in Egypt (14.7 % of the adult population is HCV seropositive) provides the unique opportunity to learn a lot about HCV [1].

The diagnosis of HCV infection is based on the detection of anti-HCV and HCV RNA. Detection of anti-HCV by immunoassay is the screening test used to evaluate HCV exposure [10]. However, among populations with low (<10 %) prevalence of HCV infection, assays for anti-HCV antibodies show high false-positive rates [11] which require confirmation with other more specific supplementary tests. Recombinant immunoblot assay is the preferred supplementary serological testing method due to its robust specificity [9, 12, 13]. According to the 2003 Centers for Disease Control and Prevention (CDC) guidelines, positive anti-HCV screening results should be confirmed using RIBA to confirm positive screening results and differentiate false positivity from true HCV exposure [14]. However, the only Food and Drug Administration (FDA) licensed supplemental anti-HCV test in the united states, Chiron RIBA HCV 3.0, has been permanently discontinued in 2011. In May of 2013, CDC issued an update on the HCV testing approach, in which it stated that the only other FDA approved supplemental tests for HCV infection are those that detect HCV viremia and recommended that future studies are needed to evaluate the performance of HCV testing without RIBA [15]. In spite of the CDC decision to remove RIBA from the diagnostic algorithm for HCV, our results indicate the necessity for continuing the use of RIBA. Egypt has the highest prevalence of HCV in the world and our results show that screening by ELISA resulted in a false positive status of 28.6 % (22/77) in HCW and 8.9 % (5/56) in presumably uninfected individuals. These cases all had a negative PCR and would have been considered as previous exposure to HCV for follow up. With the use of RIBA, their HCV status is negative with no need for follow up.

False positive results showed a highly significant increase in HCW when compared with both the presumably uninfected and chronic HCV cases. The higher percentage of false positive results of anti HCV in populations with high risk (HCW) may reflect either a past resolved infection [16] or may be the product of cross-reactivity with other viral infections such as HIV or hepatitis B [8].

Indeterminate RIBA was found in 28 cases in all the studied groups with the majority of them found in the presumably uninfected group with no viremia mainly due to a single peptide NS3 and was associated with older age. Indeterminate RIBA results with no detected viremia is consistent with results that have been previously reported [11, 17, 18] and could involve instances in which past HCV infections occurred and were eliminated without total elimination of the antibodies [17].

It has been thought that indeterminate RIBA results represent false positive reactions, however, Makuria et al., [19] have shown that the majority represent waning antibody responses in persons who have recovered from a distant HCV infection. They studied cell mediate immune responses to HCV peptides using interferon gamma elispot assay. They found out that RIBA indeterminates had strong cell mediated immune (CMI) responses, similar to those who had spontaneously recovered from HCV infection and different from those chronically infected and normal controls. They also used Luciferase Immunoprecipitation System (LIPS), and reported a stepwise diminution in antibody level from being a chronic carrier, to spontaneously recovered, to RIBA indeterminate.

The association of indeterminate RIBA results with older age is consistent with what Makuria et al. [19] has also reported in their study. They reported that RIBA-indeterminate blood donors were older than spontaneously recovered subjects or chronic HCV carriers. The older age of this group suggests that their HCV exposure might have been in the remote past allowing time for some anti-HCV antibody responses to have waned. Supporting this concept is the finding of Seeff et al. [20] who found that complete loss of antibody was shown in a retrospective-prospective study where 7 % of subjects who had anti-HCV in their original stored sample, no longer had antibody when recalled 23 years later. Interestingly, Sillanpää and coworkers [6] analysed sera from five HCV RNA and antibody positive patients during a period of 18 to 25 month. The antibody levels against the major immunogenic proteins were found to remain relatively constant. However, in three patients there were some changes in anti-HCV antibody levels, namely a weak decrease in the core and NS specific antibody levels during the follow-up period. Similar analysis by Muerhoff et al. [21] revealed that while in most cases anti-HCV antibodies remain at a constant level, there were some individuals whose antibody levels showed some fluctuation.

While waning antibody responses could explain RIBA indeterminates in HCV RNA negative individuals, we think that RIBA indeterminate results in the HCV RNA positive patients (2 chronic HCV patients), in our study, could be due to poor humoral immune response.

We found that the majority of RIBA indeterminates in presumably uninfected subjects presented antibodies that bind to HCV NS3 peptides. Similar results have been reported previously by Pereira et al. [9] who found anti-NS3 antibodies (86 %; 12/14) and anti-core antibodies (14 %; 2/14) when examining blood donors.

We focused on the study of the immunogenicity of various HCV proteins in order to reveal which viral proteins are the targets of humoral anti-HCV immune responses in humans. We examined the frequency of anti-HCV antibody responses against individual HCV proteins using RIBA test in chronic HCV, HCW and presumably uninfected subjects.

The frequency distribution of the antibody reactivity in the studied groups showed that antibodies to NS3 were lower in HCW than the other two groups and were not the most frequent antibody detected. Antibodies to NS3 have been previously associated with viral persistence. Individuals with viral persistence had higher antibody responses to NS3 as compared with individuals with apparent viral clearance from blood. Apparent viral clearance from blood was associated with a significant decrease of antibodies to NS3, independent of HCV genotype, as compared with individuals with persistent viremia [22].

In the present study, although HCV infection elicits different antibody profiles in studied group, Core 2 and NS3 were identified to separate the chronic state from HCW, while antibodies against both Core 1 and NS4 can differentiate chronic from presumably uninfected subjects. The highly significant increase of core protein in chronic HCV may be due to the fact that HCV core protein was identified as an immune-modulatory molecule suppressing T lymphocyte responsiveness through its interaction with complement receptor (gC1qR). The binding of extracellular core to gC1qR displayed on T cell surface leads to CD4+ T cell deregulation and suppression of CD8+ T cell function [23]. Moreover, HCV-core and non-structural components; NS3 and NS5A proteins, directly induce OS that results in liver damage during HCV infection [24]. These results show that antibody responses to various HCV proteins show considerable differences in frequency with certain proteins being highly immunogenic in all HCV-infected individuals while E2 were very poorly immunogenic. Beld et al. [22] stated that their findings suggest that NS3 and NS5 antibody titers may be a marker for chronicity and an alternative for monitoring efficacy of HCV therapy.

The presence of E2 in low frequency in presumably uninfected subjects and its highly significant increase in chronic and HCW subjects were concordant with a study carried out by Chen and coworkers [25] among 60 chronic HCV patients. They revealed E2 antibodies in 98 %, core in 97 %, NS3 in 88 %, NS5 in 68 % and NS4 in 48 % of the cases. Similarly, in a another study, anti-envelope protein antibodies (anti E2) were present less frequently in patients with serological viral clearance compared with those with viral persistence, showing that anti-envelope antibody titer correlates with HCV viremia [26].

The reactivity to three or more bands in the RIBA analysis was significantly increased in chronic HCV and HCW in comparison to presumably uninfected subjects. However, the number of positive bands present on the RIBA strips was not correlated with the viral load in chronic HCV. Although, Pereira et al., also reported that it was not possible to confirm any relationship between the presence of viremia and the number of bands with observed reactivity using RIBA, they found that HCV viral load is high (>850,000 IU/m) in 65 % (15/23) of the samples that showed reactivity in all four bands [9].

## Conclusion

The HCV antibody immunoblot assay (RIBA) is still necessary for the detection of false positive cases which can occur quite frequently in countries of high prevalence as Egypt. Indeterminate RIBA results indicate a waning antibody response in elderly individuals who recovered from previous or distant HCV infection. HCV infection diagnostic strategy should be modified according to anti-HCV S/CO ratio and RIBA results.

## Abbreviations

ALT: Alanine aminotransferase
CDC: Centers for Disease Control and Prevention
CMI: Cell mediated immune
FDA: Food and Drug Administration
HCV: Hepatitis C virus
HCW: Health care workers
RIBA: Recombinant immunoblot assay
